# The Influence of Diabetes on Orthodontic Treatment: A Systematic Review of the Clinical Considerations and Challenges in Response

**DOI:** 10.3390/jcm14144879

**Published:** 2025-07-09

**Authors:** Paula García-Rios, Francisco Javier Rodríguez-Lozano, Julia Guerrero-Gironés, Miguel R. Pecci-Lloret, Ricardo E. Oñate-Sánchez, Nuria Pérez-Guzmán

**Affiliations:** 1 Gerodontologý and Special Care Dentistry Unit, Morales Meseguer Hospital, Faculty of Medicine, University of Murcia, 30008 Murcia, Spain; paula.garciar@um.es (P.G.-R.); fcojavier@um.es (F.J.R.-L.); julia.guerrero@um.es (J.G.-G.); reosan@um.es (R.E.O.-S.); nuria.perez5@um.es (N.P.-G.); 2 Biomedical Research Institute of Murcia Pascual Parrilla–IMIB, 30120 Murcia, Spain

**Keywords:** orthodontic response in diabetics, diabetes microenvironment and orthodontics, orthodontic tooth movement in diabetic patients, metabolic influence on key parameters affecting orthodontic treatment, hyperglycemia-induced bone remodeling

## Abstract

**Background/Objectives:** Diabetes mellitus is defined as a group of metabolic diseases characterized by chronically elevated blood glucose levels. This condition influences the course of orthodontic treatment, as it affects various clinical aspects of the patient that must be taken into consideration prior to initiation. Therefore, achieving adequate control and management of diabetic patients undergoing orthodontic therapy is essential. This article presents a qualitative synthesis of studies addressing how diabetes affects orthodontic treatments, emphasizing the importance of understanding the necessary considerations prior to initiating treatment and how to manage potential complications. **Methods:** This systematic review was conducted in accordance with the PRISMA (Preferred Reporting Items for Systematic Reviews and Meta-Analyses) guidelines. A database search was performed on 5 May 2025, in PubMed, Scopus, Scielo, and The Cochrane Library, using terms related to “diabetes mellitus” and “orthodontic treatments”. Studies meeting the search criteria were included, particularly those that were published in the past ten years and reported on the influence of diabetes on orthodontic treatment. The quality of the case–control studies was assessed using the Newcastle–Ottawa Scale (NOS); for cross-sectional studies, the Joanna Briggs Institute (JBI) critical appraisal checklist was used; and for experimental studies, the SYRCLE’s Risk of Bias Tool was applied. **Results:** Fourteen studies ultimately met the inclusion criteria. The evidence showed that diabetes increases gingival bleeding due to elevated levels of advanced glycation end-products (AGEs) and pro-inflammatory cytokines; reduces the efficiency of tooth movement; increases root resorption and affects bone remodeling; and compromises both periodontal and pulpal responses, thereby hindering tissue regeneration. It was also observed that the use of insulin or antidiabetic agents such as metformin may partially mitigate these adverse effects. **Conclusions:** This systematic review reveals a clear relationship between diabetes and various clinical aspects that influence the progression of orthodontic treatments. Nonetheless, further studies are needed to better understand the impact of this systemic condition on dental treatment outcomes.

## 1. Introduction

Diabetes mellitus refers to a group of heterogeneous metabolic disorders characterized by elevated blood glucose levels; this condition is known as hyperglycemia. Therefore, classic symptoms, such as polyuria, polydipsia, fatigue, weight loss, and even visual disturbances and an increased susceptibility to infections, may occur [1]. Currently, the prevalence of this condition has risen, due in part to the recent increase in life expectancy in the general population and the implementation of earlier diagnostic strategies. Epidemiological data reveal a similar prevalence between men and women, with a higher incidence in urban areas compared to rural regions, and an uneven distribution between high- and low-income countries [2,3]. Its diagnosis is established through the measurement of multiple elevated blood glucose levels, regardless of the patient’s age or sex. Thus, a patient is considered diabetic when presenting with the following values: random plasma glucose ≥ 200 mg/dL; fasting plasma glucose (8–12 h of fasting) ≥ 126 mg/dL; oral glucose tolerance test (OGTT) ≥ 200 mg/dL; and glycated hemoglobin (HbA1c) ≥ 6.5% [4]. This pathology is mainly classified into four types: type 1 and type 2 diabetes, gestational diabetes, and other specific types of diabetes such as those related to exocrine pancreatic diseases. Type 1 diabetes results from impaired insulin secretion due to the destruction of pancreatic beta cells, generally leading to an absolute insulin deficiency. Also classified as type 1 diabetes are cases that are caused by either immune checkpoint inhibitors or latent autoimmune diabetes in adults (LADA), which can develop at an older age [1,4,5]. On the other hand, type 2 diabetes is manifested due to a reduction in insulin action along with the progressive loss of beta cell function [1,5]. Physical inactivity and a high-calorie, unbalanced diet are the most important risk factors in triggering type 2 diabetes, whereas a genetic predisposition and the presence of diabetes-associated antibodies are factors that would promote the onset of type 1 diabetes [6]. Table 1 below describes the most relevant differential diagnostic considerations between these two entities.

After explaining the characteristics related to this metabolic disease, it is important to understand how it can be prevented and treated. The prevention of type 1 diabetes can be approached at three different levels: primary prevention before the onset of immune activity against beta cells, which would be carried out through either vaccine administration or immune regulation induced by the microbiota; secondary prevention in cases where normoglycemia exists but there are metabolic parameters indicating a high risk for diabetes development, which could consist of combined therapies with immunomodulatory and anti-inflammatory drugs; and tertiary prevention aimed at increasing beta cell function in cases of already existing type 1 diabetes, although there are currently no therapies available for this purpose [1]. Type 1 diabetic patients are “insulin-dependent”, meaning that they require insulin therapy to survive [2]. The amount of insulin administered to the patient will vary depending on their body weight and composition, physical activity, menstrual cycle, and insulin sensitivity at different ages. Over time, new insulins and administration techniques have been developed to optimize therapy, aiming to mimic the physiological secretion of insulin both in the onset time and duration of action [7].

On the other hand, regarding the prevention of type 2 diabetes, emphasis is placed on implementing lifestyle changes, such as smoking cessation, good sleep hygiene, maintaining a balanced diet, and engaging in regular moderate physical activity. As for the treatment options, metformin is the most widely used drug, but there are others such as alpha-glucosidase inhibitors, thiazolidinediones, or glucagon-like peptide-1 receptor agonists (GLP-1s) [1,8]. Additionally, some type 2 diabetic patients require complementary insulin therapy to achieve better glycemic control [2].

Given the chronic inflammatory state and metabolic dysregulation associated with diabetes mellitus, it is reasonable to hypothesize that this systemic condition may interfere with the biological mechanisms underpinning orthodontic tooth movement [6]. Tooth movement in response to orthodontic forces relies heavily on a finely regulated balance of bone resorption and formation, orchestrated through periodontal ligament remodeling and a transient inflammatory response. However, in individuals with diabetes, particularly those with poor glycemic control, this balance may be disrupted due to persistent hyperglycemia, increased oxidative stress, and the accumulation of advanced glycation end-products (AGEs) [8].

Hyperglycemia not only affects systemic metabolism; it also profoundly alters local microbial ecosystems, particularly the oral microbiota. Excess glucose in saliva, combined with a reduced host defense capacity and systemic inflammation, promotes dysbiosis, which increases the prevalence of gingivitis, periodontitis, and other oral infections in individuals with diabetes. Consequently, there is a need to implement personalized and minimally invasive protocols aimed at preserving oral eubiosis. Evidence indicates that quarterly programs focused on reducing bacterial load—through professional hygiene, education in brushing techniques, and the use of probiotics, paraprobiotics, and postbiotics—can significantly decrease the prevalence of periodontal pathogens such as Prevotella intermedia, Campylobacter rectus, and Porphyromonas gingivalis [9]. Moreover, several clinical trials—especially in patients with type 2 diabetes—support that supplementation with multistrand probiotics (such as Lactobacillus acidophilus and Streptococcus thermophilus for 6–12 weeks) improves parameters such as fasting blood glucose, HbA1c, and the HOMA-IR index. Although the magnitude of the effect depends on the strain, dose, and duration, these findings suggest a metabolic benefit that complements pharmacological therapies such as insulin or metformin. On the other hand, evidence regarding the use of probiotics in patients with type 1 diabetes remains limited [10].

As previously mentioned, diabetes mellitus is a metabolic disease with a high prevalence; therefore, it is essential to consider the specific factors involved in orthodontic treatment for patients with diabetes, as this systemic condition can significantly affect the progression and outcomes of dental procedures. Understanding these implications enables healthcare professionals to anticipate potential complications and manage orthodontic treatment more effectively.

The objective of this systematic review was to critically analyze recent scientific evidence regarding the influence of diabetes mellitus on clinical aspects such as bone remodeling, periodontal response, and tooth mobility during orthodontic treatment. Additionally, this review aims to identify the main risk factors and associated complications in diabetic patients undergoing orthodontic therapy.

## 2. Materials and Methods

This systematic review was conducted in accordance with the PRISMA 2020 guidelines, an acronym for “Preferred Reporting Items for Systematic Reviews and Meta-Analyses” [11]. This review was also registered in the PROSPERO database (International Prospective Register of Systematic Reviews) under the registration number CRD420251046209.

In carrying out this systematic review, a set of inclusion and exclusion criteria was considered. Accordingly, studies that met our search terms and were published between 2015 and 2025, reporting on the influence of diabetes on orthodontic treatments, were included.

Conversely, articles that examined patients without diabetes or not meeting the inclusion criteria were excluded.

The PICO model was employed to establish the inclusion criteria: population/problem (P): patients with diabetes mellitus (both type 1 and type 2); intervention (I): orthodontic treatment; comparison/control (C): patients without diabetes; and outcome (O): clinical response to orthodontic treatment (efficacy of tooth movement, consequences of diabetes on dental movement, and the impact of this condition on the surrounding dental structures, which in turn influences the response to orthodontic treatment). Thus, the PICO question is as follows: How does diabetes mellitus (P) affect orthodontic treatment (I) in terms of clinical response and complications (O) compared to patients without diabetes (C)?

### 2.1. Search Strategy

The databases PubMed, Scopus, SciELO, and The Cochrane Library were employed to conduct an exhaustive search for relevant articles. From these, studies containing relevant information on the influence of diabetes on orthodontic treatment were gathered. The search was carried out on 5 May 2025 (Table 2).

To identify the necessary search terms, the Medical Subject Headings (MeSH) thesaurus was utilized. Terms related to “diabetes” included “diabetes”, “diabetes mellitus”, “diabetic”, and “hyperglycemia”. Terms related to “orthodontic treatment” included “orthodontic”, “orthodontics”, “orthodontic treatment”, “braces”, and “orthodontic therapy”. Finally, terms related to “clinically or physiologically relevant consequences” included “tooth movement”, “orthodontic force”, “bone remodeling”, “periodontal response”, “treatment response”, “outcome”, “clinical considerations”, and “inflammation”.

To relate the terms, the Boolean operators “AND” and “OR” were used. Table 2 displays the results obtained from the article search across the databases.

### 2.2. Study Selection

Following this procedure, the articles retrieved from the search were imported into the Mendeley reference manager (Elsevier) to identify and remove duplicates. Subsequently, a preliminary selection process was carried out based on the title and abstract of the studies, according to whether they met the inclusion and exclusion criteria. Finally, to determine the eligibility of the selected studies, full-text reading and analysis were conducted.

### 2.3. Data Extraction

The following categories were extracted for each article: the author and year of publication, the type of study included, the number and age of participants, the aspects analyzed, the presence of complications, the presence or absence of a comparative analysis between diabetic and healthy patients undergoing orthodontic treatment, and the conclusions drawn from the study. Both the study selection and data extraction processes were conducted independently by two reviewers (P.G.R. and M.R.P.-L.). In cases of disagreement, consensus was reached through discussion, and, if necessary, a third reviewer (J.G.-G.) was consulted.

### 2.4. Quality Analysis

This systematic review includes two case–control studies, one cross-sectional study, and eleven experimental studies using animal models. The quality assessment of the included studies was conducted using three tools: the Newcastle–Ottawa Scale (NOS) [12], the Joanna Briggs Institute (JBI) Critical Appraisal Checklist [13], and the SYRCLE’s Risk of Bias Tool [14]. The NOS is used for evaluating case–control and cohort studies, whereas the JBI checklist is employed for assessing cross-sectional studies. The Newcastle–Ottawa Scale assigns “stars” based on whether specific criteria are met within three domains, which vary depending on the type of study being assessed. For cohort studies, the evaluated domains are selection, comparability, and outcome. For case–control studies, the third domain is exposure instead of outcome. A maximum of one star can be awarded per domain, except for the comparability domain, which may receive up to two stars. Studies receiving between 7 and 9 stars were classified as having a low risk of bias; those with 4 to 6 stars were considered to have a moderate risk of bias, and those with 0 to 3 stars were classified as having a high risk of bias. The JBI Critical Appraisal Checklist was used to assess the methodological quality of cross-sectional studies, based on the criteria recommended for this study design. Eight criteria were used to determine the methodological rigor and the extent to which the design, conduct, and analysis of the study addressed potential sources of bias. Each criterion was rated as “yes”, “no”, “unclear”, or “not applicable”. Articles were classified as having a low risk of bias if they met 6 to 8 criteria, moderate risk if they met 4 or 5 criteria, and high risk if they met 0 to 3 criteria. On the other hand, the SYRCLE’s Risk of Bias Tool (developed by the Systematic Review Centre for Laboratory animal Experimentation) was used to assess the risk of bias in animal experimental studies. This tool evaluates 10 items grouped into 6 domains: selection bias, performance bias, detection bias, attrition bias, reporting bias, and other sources of bias. Each item is assessed as “yes”, “no”, or “unclear”. Studies were classified as having a low risk of bias if they met 7 or more items and had no more than one “no”; moderate risk if they met 4 to 6 items and had no more than two or three “no” responses; and high risk if they met three or fewer items or had four or more “no” ratings. To be included in the review, each article had to meet at least 50% of the established criteria. Two reviewers (P.G.R. and F.J.R.-L.) independently assessed the studies and assigned final scores, which were compared to identify discrepancies. Any disagreements in evaluations were resolved through consensus.

## 3. Results

### 3.1. Study Selection and Flow Diagram

**Figure 1:**
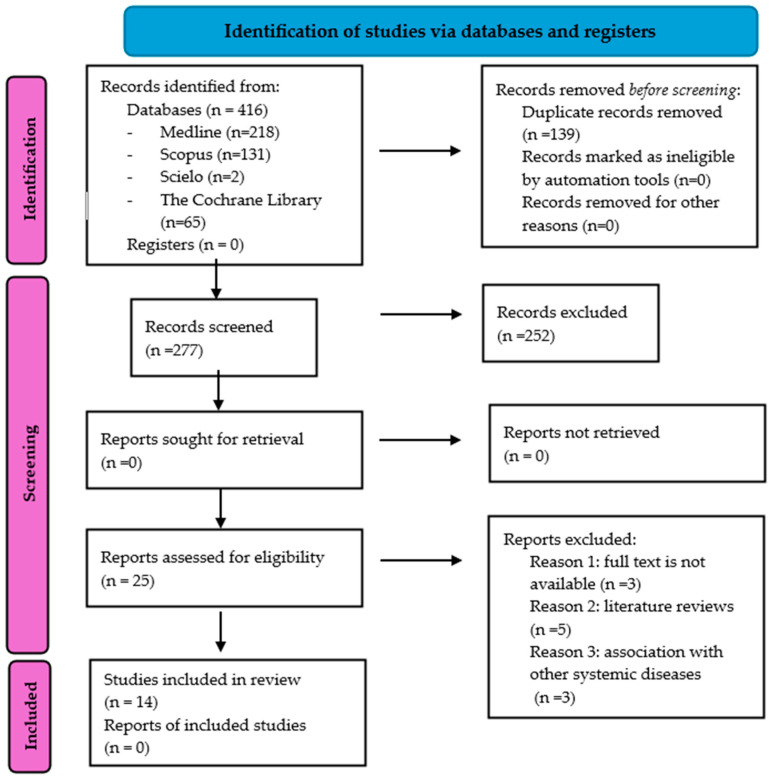
Flow diagram.

Figure 1 displays the results of the study selection process. A comprehensive search for articles was conducted across various databases, yielding a total of 416 references. Of these, 218 were from PubMed (Medline), 131 were from Scopus, 2 were from SciELO, and 65 were from the Cochrane Library. Subsequently, 139 duplicate studies were removed using the reference manager Mendeley, leaving 277 articles to be assessed based on their titles and abstracts. Later, 252 references were excluded for not meeting the inclusion criteria, as they addressed the influence of diabetes on other oral health aspects unrelated to orthodontic treatments or did not include patients with type 1 or type 2 diabetes mellitus. As a result, only 25 articles were read in full, and 11 more studies were excluded for various reasons: full text not available (*n* = 3), references classified as reviews (*n* = 5), and studies involving patients with diabetes who also presented with other systemic diseases (*n* = 3). Consequently, a total of 14 articles were selected as they met the established inclusion criteria and provided relevant information on the influence of diabetes on orthodontic treatments.

### 3.2. Data Extraction

Types of Studies

Two case–control studies, one cross-sectional study, and eleven experimental studies using animal models were included, as shown in Table 3.

### 3.3. Quality Analysis

**Figure 2:**
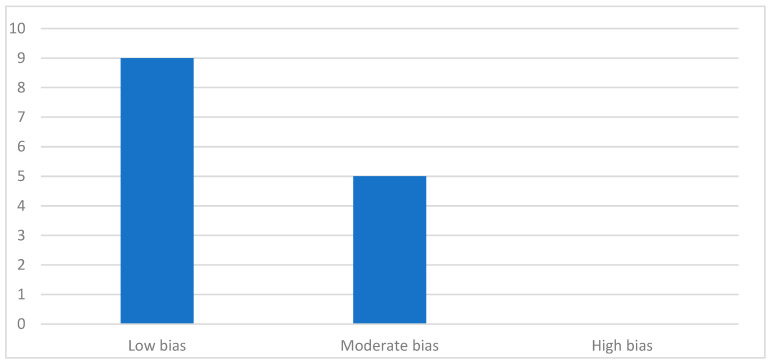
Distribution of studies according to bias [29,30,31,32].

The results of the quality assessment, based on the NOS, JBI, and SYRCLE guidelines, are presented in Table 4, Table 5, and Table 6, respectively. Of the 14 articles included in this review, nine were identified as having a low risk of bias, and the remaining ones were described as having a moderate risk of bias. Therefore, no studies were classified as having a high risk of bias. A summary of this is shown in Figure 2.

## 4. Discussion

Orthodontic tooth movement is a complex biological process that requires a tightly regulated balance between bone resorption and formation. This remodeling is orchestrated by mechanical forces and mediated by a transient, controlled inflammatory response. However, in patients with diabetes mellitus, this physiological equilibrium may be significantly altered. It is hypothesized that chronic hyperglycemia disrupts this process through the sustained elevation of pro-inflammatory mediators and oxidative stress, ultimately impairing periodontal tissue remodeling [29,30,31,32,33].

The present systematic review establishes that diabetes mellitus significantly affects various biological and clinical aspects during orthodontic treatment, with bone remodeling and the inflammatory response being the most frequently impacted processes [15,16,17,18,19,20]. Other relevant effects include root resorption [21], pulpal changes [25], the overall impact on orthodontic tooth movement [22,23,24], increased bleeding, and periodontal involvement [15,16,17,18]. The findings support the hypothesis that diabetes influences orthodontic treatments; however, methodological variability regarding the study designs and the specific clinical aspects investigated underscores the need for further research to confirm these relationships [24].

**Inflammatory Response**

The reviewed evidence indicates that diabetes mellitus exacerbates the inflammatory response during orthodontic treatment. Firstly, the study by Ying et al. [15] explores the cellular and molecular mechanisms by which diabetes, specifically type 2, affects the inflammatory response during orthodontic therapy. Using an animal model, this study demonstrated that the accumulation of advanced glycation end-products (AGEs) in rats with induced type 2 diabetes interferes with the osteogenic differentiation of periodontal ligament stem cells subjected to orthodontic forces. This process is partly due to reduced levels of the enzyme KDM6B, which is an epigenetic regulator involved in the transcription of osteogenic markers. As a result, there is a diminished capacity for bone formation and increased tissue fragility, which compromises the effectiveness of tooth movement in diabetic patients [15].

Similar findings have been reported in clinical studies involving patients with type 2 diabetes undergoing orthodontic treatment, such as those by Alshahrani et al. [16], Alqerban et al. [17], and Kamran et al. [18] These articles consistently report that diabetic patients exhibit significantly higher concentrations of AGEs and pro-inflammatory cytokines in their gingival crevicular fluid compared to healthy subjects, which is associated with increased gingival bleeding and an exacerbated inflammatory response. These effects lead to greater destruction of alveolar bone and soft tissue in patients with type 2 diabetes [16,17,18]. Furthermore, the study by Kamran et al. [18] clarifies that the inflammatory profile progressively worsens from the prediabetic state to overt diabetes. Collectively, these clinical studies reinforce the hypothesis that diabetes induces a persistent pro-inflammatory state in the periodontal environment during orthodontic treatment, thereby increasing the risk of periodontal and tissue complications [16,17,18].

**Bone Remodeling**

Bone remodeling is another key clinical aspect affected by diabetes mellitus during orthodontic treatment. Most of the findings in this area come from experimental studies using animal models, which show that both type 1 and type 2 diabetes disrupt the balance between bone formation and resorption. In the study by Alja Plut et al. [19], it was demonstrated that diabetic rats exhibited a significant reduction in alveolar bone formation and an increase in bone resorption in response to orthodontic forces, compared to healthy controls. This imbalance is attributed to a decreased osteoblastic surface and increased osteoclastic activity, although this process did not affect the expected tooth movement [19].

Additionally, Wang et al. [20] evaluated the impact of insulin-like growth factor 1 (IGF-1) on bone remodeling in type 1 diabetic rats undergoing orthodontic treatment. Their findings established that IGF-1 administration mitigated the negative effects of diabetes by accelerating tooth movement, promoting new bone formation, reducing the number of osteoclasts, and inhibiting the secretion of inflammatory factors. This suggests that certain pharmacological interventions may alleviate diabetes-induced bone alterations and thus promote a more favorable response to orthodontic treatment [20].

In summary, both experimental and clinical findings indicate that diabetes mellitus alters the bone remodeling process during orthodontic treatment, increasing the risk of bone loss and associated complications. However, the translational relevance of the results obtained from animal models should be interpreted with caution. Therefore, further clinical trials in humans are needed to confirm these findings [14,19,20].

**Root Resorption**

Root resorption during orthodontic treatment can be influenced by the presence of diabetes mellitus. Most of the evidence regarding this phenomenon comes from experimental studies in animal models. In the study by Arita et al. [21], the effect of diabetes and glycemic control on root resorption in rats subjected to orthodontic forces was evaluated. Their findings indicated that untreated diabetic rats exhibited less root resorption and tooth movement compared to healthy controls and diabetic rats receiving insulin treatment. However, the values observed in healthy animals approximated those obtained in diabetic rats with good glycemic control.

In summary, experimental evidence suggests that diabetes can modify the process of root resorption during orthodontic treatment, with glycemic control being a determining factor in the magnitude of this effect. However, the clinical significance of these findings still needs to be confirmed with clinical trials involving patients [21].

**Orthodontic Tooth Movement and Organization of Periodontal Ligament Collagen Fibers**

Orthodontic tooth movement and the organization of collagen fibers in the periodontal ligament are processes closely related to bone remodeling and the inflammatory response, both of which are altered in the presence of diabetes mellitus. Most of the available evidence comes from experimental studies in animal models.

Regarding tooth movement, results from animal models are contradictory. Studies such as those by Arita et al. [21] and Milton Santamaría et al. [22] have demonstrated a reduction in the magnitude of tooth movement under diabetic conditions, which is attributed to alterations in bone remodeling and the disorganization of periodontal fibers [21,22]. However, other studies, such as those by Lopes Ferreira et al. [23] and Ascensión Vicente et al. [24], have observed an increase in tooth movement in diabetic animals, particularly in the presence of periodontitis, which may be related to greater bone resorption and the destruction of the periodontal ligament. These contradictions may be due to differences in the experimental model design, the type of diabetes induced, the magnitude and duration of the applied forces, and the presence of other systemic or local conditions [24].

Furthermore, studies by Milton Santamaría et al. [22] and Ascensión Vicente et al. [24] have shown that diabetes, especially when uncontrolled, can lead to greater disorganization of collagen fibers in the periodontal ligament during the application of orthodontic forces [22,24]. Milton Santamaría et al. [22] observed that, in addition to poor collagen fiber organization, there is a more intense inflammatory process due to an increase in inflammatory cells and advanced glycation end-products (AGEs) [22]. The study by Ascensión Vicente et al. [24] further provided information on the activity of oxidative stress markers in normoglycemic, untreated diabetic, and insulin-treated diabetic rats. These levels were higher in untreated diabetic rats, while insulin-treated rats and healthy controls showed similar values. This evidence suggests that glycemic control can partially reverse the diabetes-induced alterations in the periodontal ligament [24].

**Pulpal Changes**

Pulpal changes associated with diabetes mellitus during orthodontic treatment have been studied in animal models. In this context, the study by Alves do Nascimento et al. [25] evaluated the pulpal response in diabetic rats subjected to orthodontic tooth movement. It was found that the dental pulp of diabetic animals exhibited greater inflammatory cell infiltration and an increased number of fibroblastic cells compared to healthy controls. These findings indicate a reduced adaptive and reparative capacity of the pulpal tissue under diabetic conditions [25].

**Effects of Medication**

The pharmacological management of diabetes mellitus can influence the biological response of patients undergoing orthodontic treatment. Most of the evidence on this aspect comes from experimental studies in animal models, in which the effects of various antidiabetic drugs—such as insulin, metformin, and DPP-4 (dipeptidyl peptidase-4) inhibitors—on bone remodeling, periodontal tissue alterations, and the extent of orthodontic tooth movement have been evaluated.

In this context, the study by Sun et al. [26] analyzed the effect of metformin, one of the drugs used in the treatment of type 2 diabetes, on bone and periodontal responses during orthodontic tooth movement. Untreated diabetic rats exhibited a significant increase in the number and activity of osteoclasts, as well as a decrease in the expression of osteoblastic markers and osteocyte function, resulting in greater bone resorption and accelerated tooth movement. However, the administration of metformin partially reversed these negative effects by reducing the number of osteoclasts and improving osteoblastic function and the expression of mineralization-related proteins, such as alkaline phosphatase (ALP) and dentin matrix protein-1 (DMP-1). As a result, tooth movement was normalized, and the integrity of the alveolar bone and periodontium was preserved [26].

Similarly, Ever Elías Mena et al. [27] reported that the combination of insulin and metformin provided greater preservation of periodontal tissues and better control of osteoclastic activity than insulin therapy alone [27].

On the other hand, Qi et al. [28] evaluated the effect of DPP-4 inhibitors, drugs used in the treatment of type 2 diabetes, and found that their administration in animal models reduced orthodontic tooth movement and root resorption, possibly due to decreased levels of osteoclasts and odontoclasts. This suggests that the use of certain antidiabetic agents may modulate the biological response to orthodontic treatment, reducing bone resorption or tooth movement [28].

Animal models have been instrumental in understanding the biological mechanisms by which diabetes mellitus affects the orthodontic response; however, they present certain limitations. Experimental models in rats and mice allow for the control of variables and the analysis of cellular and molecular processes, but there are physiological and anatomical differences compared to humans. Additionally, the experimental induction of diabetes and the application of orthodontic forces in animals often require different intensities and durations than those encountered in actual clinical conditions. Therefore, although findings from animal models provide valuable information, their direct applicability to clinical practice should be interpreted with caution. It is important to validate these results through well-designed clinical studies in humans [14].

Although the current evidence regarding orthodontic treatment in diabetic patients is limited, it is possible to suggest practical recommendations based on the available studies. Prior to initiating orthodontic treatment, a thorough medical evaluation should be conducted to ensure optimal glycemic control. This necessitates collaboration with the patient’s endocrinologist or primary care physician to monitor systemic health throughout the orthodontic process [34]. Furthermore, frequent monitoring of both periodontal status and tooth movement during treatment is required, as the risk of periodontal complications and tissue alterations is higher in diabetic patients. The use of light orthodontic forces and gradual activation protocols may help to minimize adverse effects [35,36].

Looking ahead, it is important to adopt a proactive approach in the management of diabetic patients undergoing orthodontic treatment. The establishment of regular monitoring protocols and multidisciplinary follow-up is recommended to enable early detection of any alterations in periodontal, bone, or pulpal tissues. Additionally, the implementation of preventive strategies, such as the use of natural substances (probiotics, paraprobiotics, and postbiotics) aimed at maintaining oral eubiosis and reducing complications associated with dysbiosis, may be beneficial in this context. All these measures should be accompanied by appropriate patient education, both regarding the maintenance of strict oral hygiene and glycemic control during orthodontic treatment, as these factors are essential for achieving favorable outcomes [9,37].

Nevertheless, high-quality clinical research is needed to establish standardized protocols and to clarify the most effective and safe orthodontic approaches for diabetic patients [34,35].

This review, like other articles published on the subject, presents several limitations that must be considered when interpreting the results. Firstly, not all the studies included in this review analyze the same clinical aspects influenced by the presence of diabetes in patients undergoing orthodontic treatment, which hinders direct comparisons among them. Moreover, there are significant methodological differences between the included studies, such as the type of diabetes evaluated (type 1 or type 2), the animal species analyzed and the induction models employed in the experimental studies, and the magnitude and duration of the orthodontic forces applied, as well as the medication administered (metformin, insulin, or DPP-4 inhibitors). All of these factors, together with the diversity of the experimental protocols and the type of orthodontic appliance used, further complicate interpretation of the findings and, consequently, limit the internal validity of the conclusions. Additionally, to keep the review as up to date as possible, only studies published in the last decade and in English or Spanish were included. For all these reasons, future systematic reviews should address these sources of heterogeneity in greater detail, to provide more robust and generalizable evidence.

## 5. Conclusions

In conclusion, the available evidence confirms that diabetes mellitus influences several key clinical aspects during orthodontic treatment, with bone remodeling and the inflammatory response being the most frequently reported in the scientific literature. Other relevant effects, such as root resorption and pulpal changes, are less commonly observed.

It is essential to emphasize the importance of maintaining adequate glycemic control in diabetic patients undergoing orthodontic treatment. Furthermore, an interdisciplinary approach and regular monitoring throughout orthodontic therapy are recommended to reduce complications and optimize outcomes.

Notably, certain pharmacological treatments, such as insulin, metformin, and DPP-4 inhibitors, may modify the biological response of diabetic patients during orthodontic treatment, in some cases attenuating the bone remodeling and inflammation that are characteristic of this metabolic disease. Therefore, selecting and adjusting the patients’ pharmacological therapy represents a key component of comprehensive orthodontic management of diabetic patients.

## Figures and Tables

**Table 1:** Characteristic differential considerations between type 1 and type 2 diabetes.

Criterion	Type 1 Diabetes	Type 2 Diabetes
**Etiology**	Autoimmune genetic predisposition.	Genetic predisposition, multifactorial.
**Prevalence**	<10% of cases.	Common, >90% of cases.
**Age of Onset**	Mainly during childhood or adolescence, except for LADA.	Mainly at older age, although increasingly earlier onset.
**Body Weight**	Mostly normal.	Mostly overweight.
**Symptoms**	Common.	Less common. Slow onset and often accompanied by secondary conditions.
**Tendency for Diabetic Ketoacidosis**	Yes.	None or mild.

**Table 2:** Search strategy.

Databases	Search Field	Results
Medline (PubMed)	1# “diabetes”, “diabetes mellitus”, “diabetic”, “hyperglycemia”	106,373
	2# “orthodontic”, “orthodontics”, “orthodontic treatment”, “braces”, “orthodontic therapy”	10,584
	3# “tooth movement”, “orthodontic force”, “bone remodeling”, “periodontal response”, “treatment response”, “outcome”, “clinical considerations”, “inflammation”	338,644
	**1# AND 2# AND 3#**	**218**
SCOPUS	1# “diabetes”, “diabetes mellitus”, “diabetic”, “hyperglycemia”	1,055,158
	2# “orthodontic”, “orthodontics”, “orthodontic treatment”, “braces”, “orthodontic therapy”	68,498
	3# “tooth movement”, “orthodontic force”, “bone remodeling”, “periodontal response”, “treatment response”, “outcome”, “clinical considerations”, “inflammation”	326
	**1# AND 2# AND 3#**	**131**
Scielo	1# “diabetes” fueron: “diabetes”, “diabetes mellitus”, “diabetic”, “hyperglycemia”	15,684
	2# “orthodontic”, “orthodontics”, “orthodontic treatment”, “braces”, “orthodontic therapy”	613
	3# “tooth movement”, “orthodontic force”, “bone remodeling”, “periodontal response”, “treatment response”, “outcome”, “clinical considerations”, “inflammation”	70
	**1# AND 2# AND 3#**	**2**
The Cochrane Library	1# “diabetes”, “diabetes mellitus”, “diabetic”, “hyperglycemia”	130,694
	2# “orthodontic”, “orthodontics”, “orthodontic treatment”, “braces”, “orthodontic therapy”	9619
	3# “tooth movement”, “orthodontic force”, “bone remodeling”, “periodontal response”, “treatment response”, “outcome”, “clinical considerations”, “inflammation”	933,269
	**1# AND 2# AND 3#**	**65**

**Table 3:** Results on the influence of diabetes on orthodontic treatments.

Author and Year	Type of Study	Number of Participants or Number of Rats and Comparison	Type of Diabetes	Age	Induction Model	Aspects Analyzed	Conclusions
Ying et al. (2024) [15]	Experimental study using animal models	Animal model: Rats. Five groups of male Wistar rats, each consisting of five samples. Comparison included.	Type 2	7-week-old rats.	Chemical induction	Influence of diabetes on the cellular and molecular mechanisms through which advanced glycation end-products are synthesized, affecting the osteogenic differentiation of periodontal ligament stem cells under orthodontic force.	Diabetes affects orthodontic treatment at the cellular and molecular level through the accumulation of advanced glycation end-products, impairing the ability of periodontal ligament stem cells to form bones.
Alshahrani et al. (2022) [16]	Cross-sectional study	50 individuals. 25 with type 2 diabetes and 25 non-diabetics. Comparison included.	Type 2	25–55 years old.	N/A	Levels of advanced glycation end-products and pro-inflammatory chemokines accumulated in the gingival crevicular fluid of type 2 diabetic patients undergoing orthodontic treatment were analyzed.	Patients with type 2 diabetes mellitus under fixed orthodontic treatment exhibit a biochemical profile in gingival crevicular fluid associated with a heightened pro-inflammatory response and increased gingival bleeding.
Alqerban et al. (2021) [17]	Case–control study	40 participants. 20 with type 2 diabetes and 20 non-diabetics. Comparison included.	Type 2	Mean age between 26 and 27 years.	N/A	Effect of type 2 diabetes on pro-inflammatory chemokine profiles and advanced glycation end-product levels in gingival crevicular fluid during orthodontic treatment.	Type 2 diabetic patients under fixed orthodontic treatment present elevated levels of pro-inflammatory chemokines and advanced glycation end-products in gingival crevicular fluid, resulting in increased bleeding and an intensified inflammatory response.
Kamran et al. (2024) [18]	Case–control study	75 subjects: 25 non-diabetic, 25 prediabetic, and 25 with type 2 diabetes. Comparison included.	Type 2	Mean age between 25 and 32 years.	N/A	Influence of diabetes on the deposition of advanced glycation end-products and pro-inflammatory cytokines in gingival crevicular fluid and saliva.	Patients with type 2 diabetes under fixed orthodontic treatment show elevated levels of pro-inflammatory biomarkers in gingival crevicular fluid, associated with greater bleeding and an exacerbated inflammatory response.
Alja Plut et al. (2015) [19]	Experimental study using animal models	Animal model: Rats. 48 rats. 24 male Wistar rats (healthy controls) and 24 male GK rats (type 2 diabetic). Comparison included.	Type 2	Age of rats not specified.	GK rat	Effects of type 2 diabetes on bone remodeling during orthodontic tooth movement. Dental movement in diabetic rats was also measured.	Diabetes influences alveolar bone remodeling, causing a reduction in bone formation and increased resorption in response to orthodontic force.
Wang et al. (2023) [20]	Experimental study using animal models	Animal model: Rats. 60 Sprague Dawley rats are divided into 3 groups (20 per group): control, diabetic, and IGF-1 group. Comparison included.	Type 1	3-month-old rats.	Chemical induction	Influence of insulin-like growth factor 1 (IGF-1) on alveolar bone remodeling during orthodontic tooth movement in diabetic rats.	Diabetes reduces tooth movement and negatively affects bone remodeling during orthodontic treatment. It also increases inflammation and osteoclastic activity. IGF-1 treatment mitigates these adverse effects by improving bone remodeling.
Arita et al. (2016) [21]	Experimental study using animal models	Animal model: Rats. 23 male Sprague-Dawley rats: 7 control, 9 diabetic, 7 diabetic + insulin. Comparison included.	Type 1	10-week-old rats.	Chemical induction	Influence of diabetes and insulin administration on tooth movement and root resorption.	Diabetes reduces the rate of tooth movement and the extent of root resorption during orthodontic treatment. Glycemic control with insulin can largely reverse these effects.
Milton Santamaria et al. (2019) [22]	Experimental study using animal models	Animal model: Rats. 40 male Wistar rats divided into 4 groups (10 per group): healthy rats with OTM; rats with periodontitis and OTM; diabetic rats with OTM; and rats with both periodontitis, diabetes, and OTM. Comparison included.	Type 1 and 2	90-day-old rats.	Chemical induction	How diabetes alters the inflammatory response of gingival tissue and alveolar bone during orthodontic movement.	Diabetes increases local inflammatory response during orthodontic movement, resulting in less efficient tooth movement compared to non-diabetic conditions.
Lopes Ferreira et al. (2018) [23]	Experimental study using animal models	Animal model: Rats. 40 male Wistar rats divided into diabetic and non-diabetic groups. Comparison included.	Type 1	90-day-old rats.	Chemical induction	Histological periodontal responses to orthodontic tooth movement in rats with induced diabetes. Also analyzed were bone loss, bone density, amount of tooth movement, bone resorption, and periodontal ligament destruction.	Diabetes has a negative effect on the supporting dental bone, leading to greater bone loss during orthodontic treatment.
Ascensión Vicente et al. (2020) [24]	Experimental study using animal models	Animal model: Rats. 60 male Sprague-Dawley rats divided into 3 groups: 20 normoglycemic, 20 untreated diabetic, and 20 insulin-treated diabetics. Comparison included.	Type 1	Adult rats (exact age not specified).	Chemical induction	Influence of diabetes, with or without treatment (simulating type 1 diabetes), on various aspects of orthodontic tooth movement: oxidative stress levels, orientation of periodontal ligament fibers, matrix metalloproteinase expression, and amount of tooth movement.	In untreated diabetic rats, orthodontic force causes increased inflammation, oxidative stress, disorganization of the periodontal ligament, and greater expression of matrix-degrading enzymes. Insulin largely reverses these effects.
Alves do Nascimento et al. (2021) [25]	Experimental study using animal models	Animal model: Rats. 40 male Wistar rats divided into 4 groups: 10 healthy controls without OTM; 10 diabetics without OTM; 10 non-diabetics with OTM; and 10 diabetics with OTM. Comparison included.	Type 1	90-day-old rats.	Chemical induction	Pulpal responses to orthodontic tooth movement in rats with type 1 diabetes mellitus.	Pulp tissue of teeth subjected to orthodontic movement in the presence of diabetes mellitus shows reduced adaptive and reparative capacity.
Sun et al. (2017) [26]	Experimental study using animal models	Animal model: Rats. 30 Wistar rats divided into 3 groups: 10 normoglycemic controls, 10 with type 2 diabetes, and 10 with type 2 diabetes treated with metformin. Comparison included.	Type 2	Age not specified.	Chemical induction	Effects of metformin, a drug used to treat type 2 diabetes, orthodontic tooth movement, alveolar bone remodeling, and root resorption.	Type 2 diabetes in rats increases osteoclast number and activity, leading to greater tooth movement under orthodontic treatment. Metformin reduces osteoclast activity, enhances osteoblast function, and restores osteocyte function, thus normalizing tooth movement.
Ever Elias Mena et al. (2019) [27]	Experimental study using animal models	Animal model: Rats. 80 male Albinus Wistar rats divided into 4 groups: normoglycemic controls, untreated type 1 diabetics, type 1 diabetics treated with insulin, and type 1 diabetics treated with insulin and metformin. Comparison included.	Type 1	8-week-old rats.	Chemical induction	Effect of metformin (used in addition to insulin) on periodontal response during orthodontic tooth movement in type 1 diabetic rats. Also evaluated movement pattern, amount of tooth movement, and alveolar bone changes.	Untreated type 1 diabetes causes severe periodontal damage and an altered pattern of tooth movement under orthodontic force. Antidiabetic treatment (insulin or insulin plus metformin) reduces this damage, producing a response like that of non-diabetic rats.
Qi et al. (2020) [28]	Experimental study using animal models	Animal model: Mice. 24 male C57BL6/J mice divided into 3 groups: OTM + DPP-4 inhibitor; OTM + phosphate-buffered saline; control. Comparison included.	Type 2	8–10 weeks old.	Chemical induction	Effects of DPP-4 inhibitors (used to treat type 2 diabetes) on tooth movement distance and root resorption.	DPP-4 inhibitors, drugs used to treat type 2 diabetes, may negatively affect orthodontic treatment by reducing tooth movement and root resorption.

**Table 4:** The quality assessment of the studies using the adapted version of NOS for case–control studies. Stars indicate the degree to which each study meets the NOS criteria. A maximum of one star can be awarded for each item in the “Selection” and “Exposure” domains, and up to two stars in the “Comparability” domain. Color coding indicates the level of risk of bias: green for low risk.

Case–Control Studies (NOS)	Selection	Comparability	Exposure	Total Score
Alqerban et al. [17]	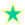 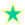 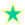	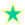 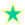	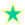 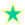	7 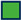
Kamran et al. [18]	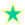 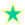 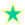	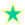 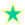	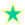 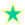	7 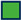

**Table 5:** JBI Checklist Evaluation. Color coding indicates the response: green for “yes”, red for “no”.

Article Title	Clear Inclusion Criteria	Subjects and Setting Described	Exposure Measured Validly	Standard Criteria for Condition	Confounding Factors Identified	Strategies to Deal with Confounding	Outcomes Measured Validly	Appropriate Statistical Analysis	Overall Appraisal	%
**Alshahrani et al. (2022) [16]**	Yes 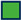	Yes 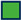	Yes 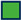	Yes 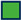	Yes 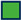	No 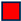	Yes 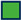	Yes 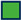	Include 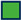	**87.5**

**Table 6:** SYRCLE’s Risk of Bias Tool. Color coding indicates the response: green for “yes”, red for “no”, and yellow for “unclear”.

Study	1. Appropriate Random Allocation	2. Similar Baseline Characteristics	3. Allocation Concealment	4. Blinding of Personal/Care Givers	5. Blinding of Outcome Assessors	6. Incomplete Data Adequately Handled	7. Selective Reporting Avoided	8. Free from Other Biases	9. Funding Without Conflict of Interest	10. Appropriate Experimental Design	Overall Risk
Ying et al. [15]	No 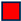	Yes 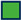	Unclear 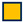	No 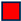	No 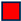	Yes 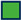	Yes 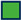	Yes 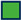	Yes 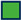	Yes 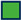	Moderate 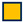
Alja et al. [19]	Yes 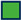	Unclear 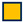	Yes 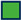	No 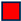	Unclear 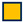	Yes 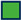	Yes 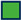	Yes 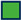	Yes 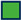	Yes 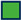	Low 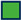
Wang et al. [20]	Yes 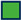	Yes 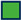	Unclear 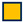	No 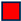	Unclear 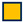	Yes 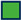	Yes 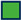	Yes 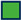	Yes 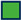	Yes 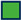	Low 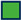
Arita et al. [21]	Yes 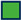	Yes 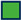	Unclear 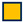	No 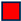	No 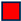	Yes 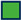	Yes 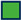	No 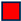	Yes 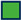	Yes 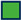	Moderate 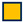
Milton Santamaría et al. [22]	Yes 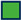	No 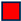	Unclear 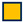	Unclear 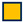	Yes 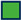	Yes 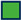	No 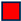	No 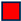	Yes 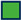	Yes 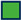	Moderate 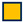
Lopes Ferreira et al. [23]	Yes 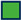	Yes 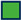	Unclear 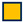	Yes 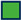	Unclear 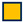	No 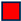	Yes 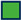	Yes 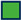	Yes 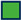	Yes 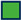	Low 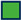
Ascensión Vicente et al. [24]	Unclear 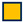	Unclear 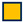	Yes 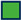	Yes 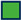	Yes 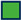	No 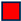	Yes 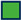	Yes 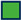	Yes 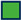	Yes 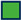	Low 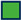
Alves do Nascimento et al. [25]	Yes 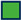	Yes 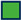	Unclear 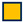	Unclear 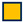	Yes 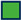	No 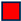	No 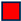	Yes 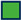	Yes 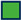	Yes 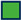	Moderate 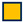
Sun et al. [26]	Unclear 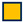	Unclear 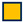	Yes 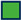	Yes 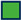	Yes 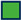	No 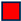	Yes 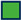	Yes 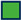	Yes 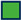	Yes 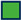	Low 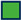
Ever Elías Mena et al. [27]	Yes 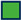	Yes 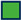	Unclear 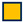	No 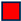	Unclear 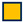	No 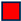	Yes 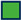	Yes 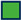	Yes 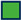	Yes 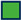	Moderate 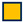
Qi et al. [28]	Unclear 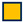	Yes 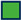	Unclear 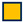	Unclear 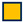	Yes 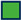	Yes 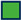	Yes 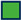	Yes 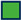	Yes 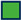	Yes 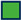	Low 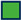
